# A Model-Based Approach for Separating the Cochlear Microphonic from the Auditory Nerve Neurophonic in the Ongoing Response Using Electrocochleography

**DOI:** 10.3389/fnins.2017.00592

**Published:** 2017-10-23

**Authors:** Tatyana E. Fontenot, Christopher K. Giardina, Douglas C. Fitzpatrick

**Affiliations:** ^1^Otolaryngology-Head and Neck Surgery, University of North Carolina, Chapel Hill, NC, United States; ^2^School of Medicine, University of North Carolina, Chapel Hill, NC, United States

**Keywords:** cochlear physiology, electrophysiology, auditory hair cells, auditory nerve, auditory nerve model, computational modeling, modeling and simulations

## Abstract

Electrocochleography (ECochG) is a potential clinically valuable technique for predicting speech perception outcomes in cochlear implant (CI) recipients, among other uses. Current analysis is limited by an inability to quantify hair cell and neural contributions which are mixed in the ongoing part of the response to low frequency tones. Here, we used a model based on source properties to account for recorded waveform shapes and to separate the combined signal into its components. The model for the cochlear microphonic (CM) was a sinusoid with parameters for independent saturation of the peaks and the troughs of the responses. The model for the auditory nerve neurophonic (ANN) was the convolution of a unit potential and population cycle histogram with a parameter for spread of excitation. Phases of the ANN and CM were additional parameters. The average cycle from the ongoing response was the input, and adaptive fitting identified CM and ANN parameters that best reproduced the waveform shape. Test datasets were responses recorded from the round windows of CI recipients, from the round window of gerbils before and after application of neurotoxins, and with simulated signals where each parameter could be manipulated in isolation. Waveforms recorded from 284 CI recipients had a variety of morphologies that the model fit with an average *r*^2^ of 0.97 ± 0.058 (standard deviation). With simulated signals, small systematic differences between outputs and inputs were seen with some variable combinations, but in general there were limited interactions among the parameters. In gerbils, the CM reported was relatively unaffected by the neurotoxins. In contrast, the ANN was strongly reduced and the reduction was limited to frequencies of 1,000 Hz and lower, consistent with the range of strong neural phase-locking. Across human CI subjects, the ANN contribution was variable, ranging from nearly none to larger than the CM. Development of this model could provide a means to isolate hair cell and neural activity that are mixed in the ongoing response to low-frequency tones. This tool can help characterize the residual physiology across CI subjects, and can be useful in other clinical settings where a description of the cochlear physiology is desirable.

## Introduction

Electrocochleography is the recording of electrical potentials produced by the cochlea in response to stimulation. It has been extensively used to evaluate peripheral auditory system physiology, and is used clinically to identify hydrops in Meniere's patients and other retrocochlear pathologies (Schmidt et al., 1974; Gibson and Beagley, 1976). It has also drawn interest for the study of auditory neuropathy spectrum disorder (ANSD, Santarelli, 2010; Rance and Starr, 2015). Recently, ECochG has been used to account for speech perception outcomes in cochlear implant (CI) recipients (Fitzpatrick et al., 2014; McClellan et al., 2014; Formeister et al., 2015) and is showing promise for detecting intraoperative trauma in CI patients (Adunka et al., 2010; Mandala et al., 2012; Radeloff et al., 2012; Calloway et al., 2014; Campbell et al., 2015; Dalbert et al., 2015, 2016; Bester et al., 2017). Liberman and colleagues, among others, have investigated various aspects of ECocG for detecting evidence of cochlear synaptopathy, or hidden hearing loss (Liberman et al., 2016). Analysis of the hair cell and neural contributions to ECochG responses recorded in CI recipients is the main objective of this study.

The responses from the cochlea to sounds consist of several distinct signals which overlap in time. The compound action potential (CAP) occurs near the onset of the response to stimuli with fast rise times, and has a purely neural source produced by the synchronous action potential produced to onsets of sound. The alternating-current (AC) component of the ECochG response is a mixture of the cochlear microphonic (CM) and auditory nerve neurophonic (ANN). The CM is produced by transducer current through stereocilia of hair cells in response to basilar membrane movement, and is thus phase-locked to all tone frequencies. The ANN is the evoked potential correlate of phase-locked responses in neural fibers, which is strong only to frequencies below ~2,000 Hz. The direct current (DC) response to tones is the summating potential (SP) which is derived from a complex mixture of hair cell (Davis et al., 1958; Dallos, 1973; Zheng et al., 1997; Durrant et al., 1998) and neural (van Emst et al., 1995; Sellick et al., 2003; Forgues et al., 2014) sources.

There are several cases where it would be useful to separate the CM from the ANN in the ongoing portion of the response to tones. These include a non-invasive way to estimate the upper limit of phase locking (Verschooten and Joris, 2014; Verschooten et al., 2015); as a screen for low frequency hearing loss (Lichtenhan et al., 2013, 2014); and to determine the proportions of hair cell and neural activity in the responses of CI recipients, which are most reliably elicited by low frequency stimuli (Choudhury et al., 2012). Historically, the ANN was considered the principal source of the 2nd harmonic (Henry, 1995; Lichtenhan et al., 2013; Chertoff et al., 2015). However, asymmetries of the transduction process also produce even harmonics in the CM (Teich et al., 1989; Santos-Sacchi, 1993; Forgues et al., 2014). The periodicity of both the CM and the ANN reflect the stimulus frequency, thus, both potentials contribute to the magnitude of the first harmonic peak (Snyder and Schreiner, 1984; Forgues et al., 2014; Verschooten et al., 2015). Masking has been used to recover the proportion of the neural response removed by adaptation, based on the idea that only neural signals show such adaptation (Snyder and Schreiner, 1984; Sparacino et al., 2000; Verschooten et al., 2015). However, this approach only quantifies the neural proportion that adapts to the masker, and cannot quantify the total amount of neural response within the signal.

The approach presented here uses discrete analytic models of the expected ANN and CM waveforms in order to separate them in the combined signal, as would be acquired in a clinical setting. By varying the proportions of expected CM and ANN, and the phases between them, we can determine the best fit for the parameters to match the recorded waveforms. To validate the approach we first show that the model is able to fit the complex waveforms recorded from human CI subjects. We then examine the parametric performance of the model using artificially mixed signals, and show results from animals before and after application of the neurotoxins kainic acid (KA), tetrodotoxin (TTX), and ouabain (OA) to the round window. Finally, the model is used to examine the CM and ANN in responses from CI recipients.

## Methods

Three data sets were used in the experimental design: human CI recipients, gerbils, and simulated signals created by varying the parameters of interest.

### Human CI recipients

All adult and pediatric patients who were scheduled for CI at University of North Carolina Hospitals in 2011–2017 were eligible to be enrolled in the study. Thus, the sample population (*N* = 285) includes the heterogeneity of conditions leading to a recommendation for a CI. Non-native English speakers, children of non-native speakers, and those undergoing revision surgery or with severe inner ear malformations (cochlear atresia, etc.) were excluded. The recordings in human CI recipients were carried out in accordance with the recommendations of Declaration of Helsinki guidelines as reviewed and approved by the Institutional Review Board at University of North Carolina. All subjects gave written informed consent in accordance with the Declaration of Helsinki. Parental consents were obtained for all pediatric subjects and assent was obtained for pediatric subjects at least 7 years old.

The recording procedures for pediatric and adult CI recipients have been previously described (Choudhury et al., 2012; McClellan et al., 2014; Formeister et al., 2015). A Biologic Navigator PRO (Natus Medical Inc., San Carlos, CA) was used for acoustic stimulation and ECochG recordings. The stimuli were delivered through an in-ear foam insert attached to a speaker (Etymotic ER3b) by a sound tube. Stimuli were alternating phase tone bursts from 250 to 4,000 Hz presented at 90 dB nHL (from 108 to 114 dB peak SPL for 250–2 kHz, 95 dB for 4 kHz). Rise/fall times were 1 ms or 1 cycle, whichever was longer. Calibration of sound levels was by a ¼″ microphone and measuring amplifier (Bruel and Kjaer, Nærum, Denmark). Distortion at these sound levels for the second harmonic was from −37 to −67 dB compared to the fundaments for frequencies of 1–2 kHz, but was −26 dB for 4 kHz. The third harmonic was < −40 dB compared to the fundamental for all frequencies.

A standard transmastoid facial recess approach was used to surgically access the round window. The recording used surface electrodes on the forehead contralateral mastoid as ground and reference electrode, respectively. The active electrode a stainless-steel monopolar probe (Neurosign; Magstim Co., Wales, UK) placed in the round window niche. The ECochG recordings were obtained immediately before CI insertion. Recording epochs were 512 points each, from 32 ms for 250–1,000 Hz (16,000 Hz sampling rate) to 10.66 ms for 2,000 and 4,000 Hz (48,000 Hz sampling rate). Filter settings were 10 Hz high-pass and low passes were 5,000 Hz for 250–1,000 Hz, and 15,000 Hz for 2 and 4 kHz.

### Recordings in gerbils

The experiments with gerbils (*Meriones unguiculatus*) were carried out in accordance with the standards of the National Institutes of Health and Committee on Care and Use of Laboratory Animals. All procedures were reviewed and approved by the Institutional Animal Care and Use Committee (IACUC) at the University of North Carolina.

Gerbils with clean middle ears had ECochG recordings using the same equipment as in the human recordings. Anesthesia, surgery, and ECochG recording procedures have been previously described (Forgues et al., 2014). Animals were sedated using sodium pentobarbital (10 mg/kg, i.p.) and anesthetized with urethane (1.5 g/kg, i.p.), Atropine was used to control respiratory secretions. The animal was maintained at 38°C using a heating pad. Needle electrodes were placed at the base of the tail and contralateral neck muscles for the ground and reference inputs, respectively. A sealed sound tube was then placed within the external auditory canal. A sealed sound tube was then placed within the external auditory canal. After surgical exposure of the round window, the Neurosign electrode was placed inside the niche. Tone bursts of 250–8,000 Hz over levels from 30 to 80 dB SPL were presented with the same stimulus/recording conditions as for the humans. Additional frequencies in some cases included 375 and 8,000 Hz; both had second and third harmonic distortion levels of < −50 dB compared to the fundamental.

The neurotoxins KA, TTX, and OA were used to obtain signals with diminished neural contribution. Different substances were used because the material was available from other experiments, and because the use of multiple compounds can help avoid the possibility of one or the other having unexpected actions on hair cells in addition to nerve fibers. KA is a glutamate analog and destroys the nerve terminals by excitotoxicity; TTX blocks sodium channels and thus removes the spiking component of the neural response, and OA inhibits the sodium pump also blocking the nerve from firing as well as further depolarizing, but without physically removing the nerve terminal. Six animals were used for each substance. The neurotoxins were applied for 1 h to the round window following baseline ECochG recordings. The toxins were dissolved in lactated Ringer's solutions for KA, and artificial perilymph for TTX and OA. The solutions were warmed to 38°C before use. The KA (Sigma USA #K0250) was 60 or 100 mM; the TTX was 15 μM (Tocris Bioscience, #1069) and the OA (Calbiochem, #4995) was 1 or 10 mM. After application the solutions was wicked from the round window and replaced with vehicle alone. The ECochG recording series was then performed again.

### Signal analysis

**Figure 1:**
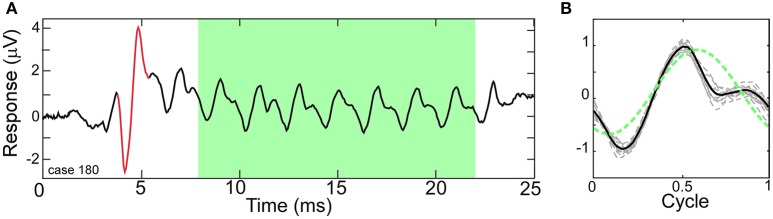
Electrocochleography (ECochG) response to a tone burst from a human CI subject. **(A)** A Human ECochG response to a 500 Hz tone burst presented in condensation phase. The ongoing portion is highlighted (green area). The CAP is shown in red. **(B)** Each cycle in the ongoing response (dashed lines) and the “average cycle” (solid line). The presence of the ANN causes distortions in the response compared to a reference sinusoid (dotted line).

Figure 1A depicts a typical ECochG response to a 500 Hz condensation-phase tone burst with the ongoing portion highlighted (green area). Within this region, the CM and ANN are mixed together, with both following the amplitude changes in the tone. Each cycle of the ongoing portion of the response was combined to produce an “average cycle” (Figure 1B). The mixture of the CM and ANN affect the distortions in the response, compared to the sinusoidal stimulus (dashed green line). This average cycle became the input that the model attempted to fit.

The time waveforms were analyzed with using fast Fourier Transforms (FFTs) and the magnitude peaks to the stimulus frequency and its harmonics were considered significant if they exceeded the noise by more than three standard deviations, as measured from three bins on either side of the peaks. Typically, the minimum detectable signal was ~20 nV after 500 repetitions (−34 dB re 1 μV).

For the human CI subjects, evidence of neural activity from CI recipients was graded based on a visual assessment of the response, including evaluation for the presence of a CAP and ANN across the frequency range (Riggs et al., 2017). Briefly, a CAP was typically detected as a negative deflection within the first few ms of the response (although some were delayed as long as 10 ms, see Scott et al., 2016; Abbas et al., 2017). The ANN was determined to be present when the average cycle deviated from a possible shape attributable to the CM alone, as further described below. The CAP and ANN were each scored over the range of 0–2, so the range of “nerve scores” was from 0 to 4. A zero for the CAP or ANN indicated no conclusive evidence of presence; one indicated present but small (in the case of the CAP), or with clear but relatively minor distortions in the average cycle (in the case of the ANN); while two indicated large (in the case of CAP) or with strong distortions (for the ANN). The shapes of the average cycle that indicated the presence of the ANN was strongly influenced by the animal work reported in part here. For examples of human CI cases with each nerve score, see Riggs et al. (2017). It was the need for an objective means of determining the presence of the ANN that prompted the development of the model reported here. The nerve score is useful as an independent means of assessing neural activity (see **Figure 11**).

### The conceptual basis of the model

**Figure 2:**
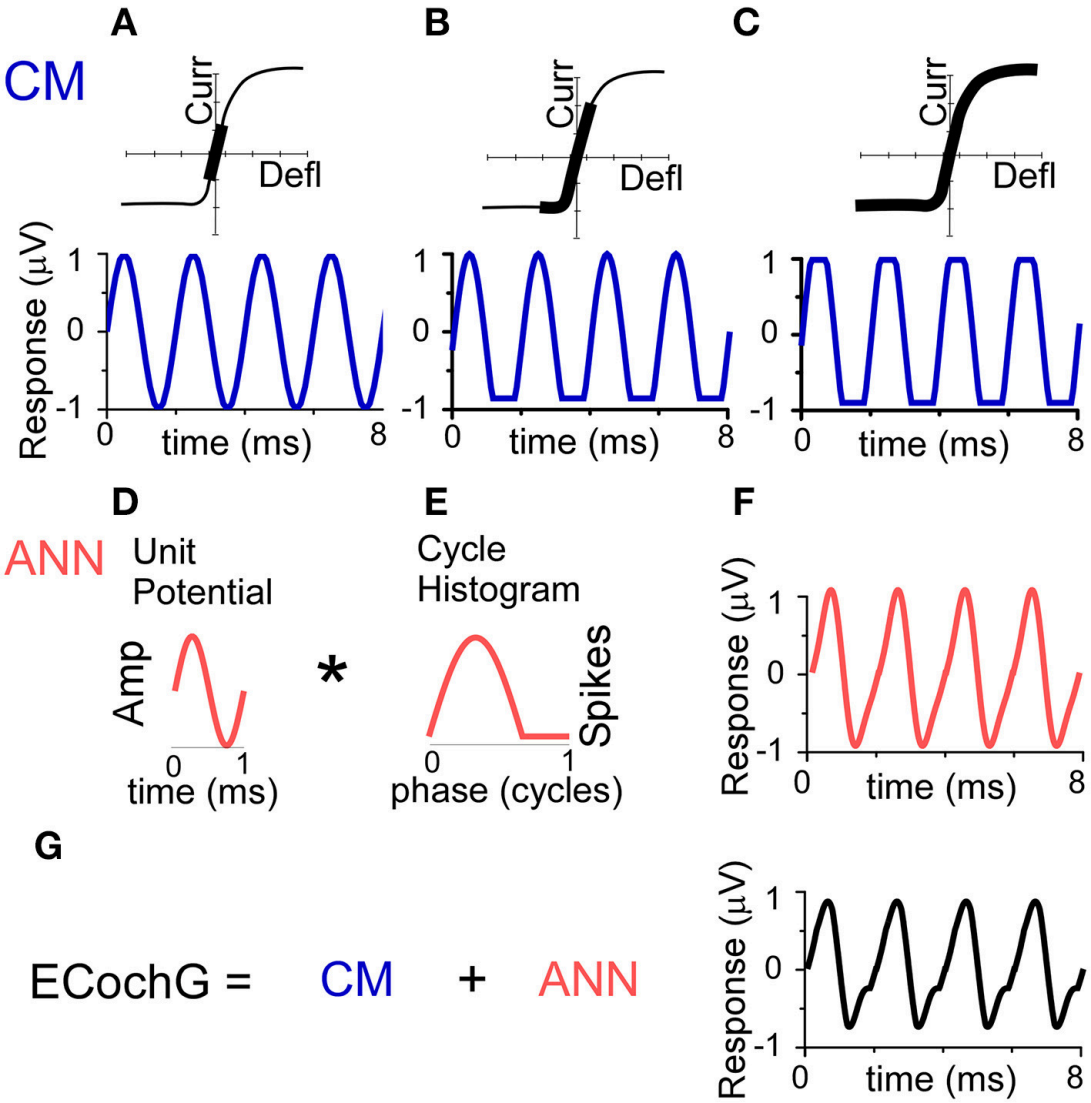
Conceptual basis of the model for the ongoing part of the ECochG response to low frequency tones. **(A–C)** The CM. To a low stimulus intensity **(A)**, the hair cell stereociliary motion and channel openings operate symmetrically within the input-output function (top, black bar), producing a sinusoidal CM response (bottom). **(B)** With increasing stimulus intensity, asymmetric saturation can occur if the operating point (average state of the channels at rest) is displaced from the center of the function (top), producing a CM saturated only to one side of motion, in this case the trough of the CM (bottom). **(C)** With a high stimulus intensity, symmetric saturation occurs with maximal deflection at both ends of the oscillation (top), creating a CM with saturation to both the peak and trough. **(D–F)** The ANN is created by the convolution (^*^) of the unit potential **(D)** and the population cycle histogram **(E)**. The unit potential is the shape of a single action potential at the round window, and the cycle histogram is the sum of action potential firing in the population of the across all responding nerve fibers. Because the cycle histogram is derived by folding the periods in the post-stimulus time histogram, this process is identical to that previously modeled to produce the CAP (see text for references). The non-linearities inherent in this process will always create a distorted version of the cyclic response **(F). (G)** The ongoing ECochG represents the sum of the CM and ANN.

The conceptual basis for the individual contributions of CM and ANN used in the model are depicted in Figure 2. The source of the CM is the transducer current through mechanosensitive channels in the stereocilia of hair cells. The input-output function of the current flow is typically modeled as an asymmetrically saturating second-order Boltzmann function (Santos-Sacchi, 1993; Sirjani et al., 2004; Ramamoorthy et al., 2007). To a low intensity stimulus (Figure 2A), the hair cell movement is within the linear range of the function producing a sinusoidal CM. To a moderate intensity stimulus (Figure 2B), the hair cell movement can saturate in one direction producing a partially rectified signal, depending on the degree of distance of the operating point, or proportion of open channels at rest, from the midpoint of the function. For a high intensity stimulus, the movement saturates in both directions of the CM waveform (Figure 2C). Thus, the CM can be represented as a sinusoid at the stimulus frequency, with two additional parameters of saturation of the peak and trough of response, to capture both asymmetric and symmetric saturation.

As with the CAP, the ANN can be described as the convolution of a unit potential (UP), which is the shape of a single action potential as it appears at the round window (Kiang et al., 1976; Prijs, 1986; Versnel et al., 1992a), and the cumulative post-stimulus time histogram, or summed histogram of all responding auditory nerve fibers (Goldstein and Kiang, 1958; Snyder and Schreiner, 1984; Chertoff, 2004). For low frequency tones, the post-stimulus time histograms of auditory nerve fibers shows cyclic firing to the positive-going half-phase of the stimulus (Rose et al., 1967). By folding across stimulus cycles, the resulting cycle histogram (CH) resembles the half-wave rectified form of the phase-locking. The curve shown (Figure 2E) has been stretched to be more than a half-cycle to simulate the spread in phase associated with inclusion of fibers at more basal positions on the basilar membrane as the intensity is varied (Kim and Molnar, 1979).

### Implementation of the model

The CM was described by Equation (1). A sinusoid (Equation 1a) was defined in time (*t*, in seconds) with frequency (*f* in Hz) equal to the stimulus frequency and amplitude (*A*_*CM*_ in μ V) and starting phase (φ_*CM*_, in cycles) as parameters. Additional parameters were upper and lower cutoffs that represented saturation of the peak and trough independently (Equation 1b). The *A*_*CM*_ was allowed to vary between 0 and 5x the maximum of the input signal. The phase boundaries were from −2 to 2 cycles. Boundaries of clipping the peak and trough were 50% of the maximum or minimum input, respectively.

(1a) $$CM_{sine}(t)=A_{CM}\times \sin\left(2\Pi \left(ft-\varphi_{CM}\right)\right)$$

(1b) $$CM\left(t\right)=\{\begin{array}{ll} UpperCutoff & \;if\;CM_{sine}(t)\;>UpperCutoff\;\\ CM_{sine}(t) & \;if\;LowerCutoff\leq \;CM_{sine}(t)\\ \; & \;\leq UpperCutoff\\ LowerCutoff & \;if\;CM_{sine}(t)<LowerCutoff \end{array}$$

To fit the neural contributions to the ongoing response, the UP was described as a single cycle of a sinusoid at 1,100 Hz. This frequency was selected based on pilot studies where values over the range of 800–1,200 Hz were tested, where 1,100 Hz provided the best fits on average. The UP has also been previously modeled using a dampened sinusoid (Chertoff, 2004) but we found that a peak in a second cycle of the UP introduced distortions not reflective of those seen in the physiological data, producing poor fits. The cycle histogram (CH), was described as a lognormal probability distribution function (Equation 2) which describes when neural spikes are most likely to fire. Probability in the CH is highest during the phase of basilar membrane motion that depolarizes hair cells, and is zero for the hyperpolarizing direction because the spike rate cannot go below zero (although spontaneous activity can be modulated; Rose et al., 1967). The width of the CH distribution curve (σ) was determined by the “SOE” parameter, which was allowed to range from 0.35 to 0.65 of the stimulus cycle. The lower limit was chosen because it is sharper than the vector strength of a typical nerve fiber over most frequencies and intensities, so a sharper cycle histogram for the population is not expected. The upper limit was chosen because there is a natural limit for SOEs greater than one cycle, because only the cyclic part of the ANN contributes to the ac component of the ongoing response as because a constant level of firing occurs as the cycle histogram from different regions overlap.

(2) $$H(t)=\frac{1}{(\sigma \sqrt{2}\pi )t}e^{\frac{-{\left(\ln t-\mu \right)}^{2}}{2\sigma^{2}}}$$

*t* = timeline of the CH, μ = period of UP, and σ = SOE

Convolution of the UP and the CH, multiplied by an ANN amplitude term, *A*_*ANN*_, was performed to yield a single cycle of ANN (Equation 3). The *A*_*ANN*_ was allowed to vary between 0 and 5 times the maximum of the input signal.

(3) $$ANN(t)=A_{ANN}\times (CH\left(t\right)*UP\left(t\right))$$

Phase shift (φ_*ANN*_) was a parameter applied to the convolved signal using MATLAB function “circshift” which discretely shifts the array circularly. It could vary over the range of −2 to 2 cycles.

The two signals were then summed to produce the model ECochG by Equation (4).

(4) $$ECochG_{model}(t)\;=ANN(t)+\;CM(t)$$

**Figure 3:**
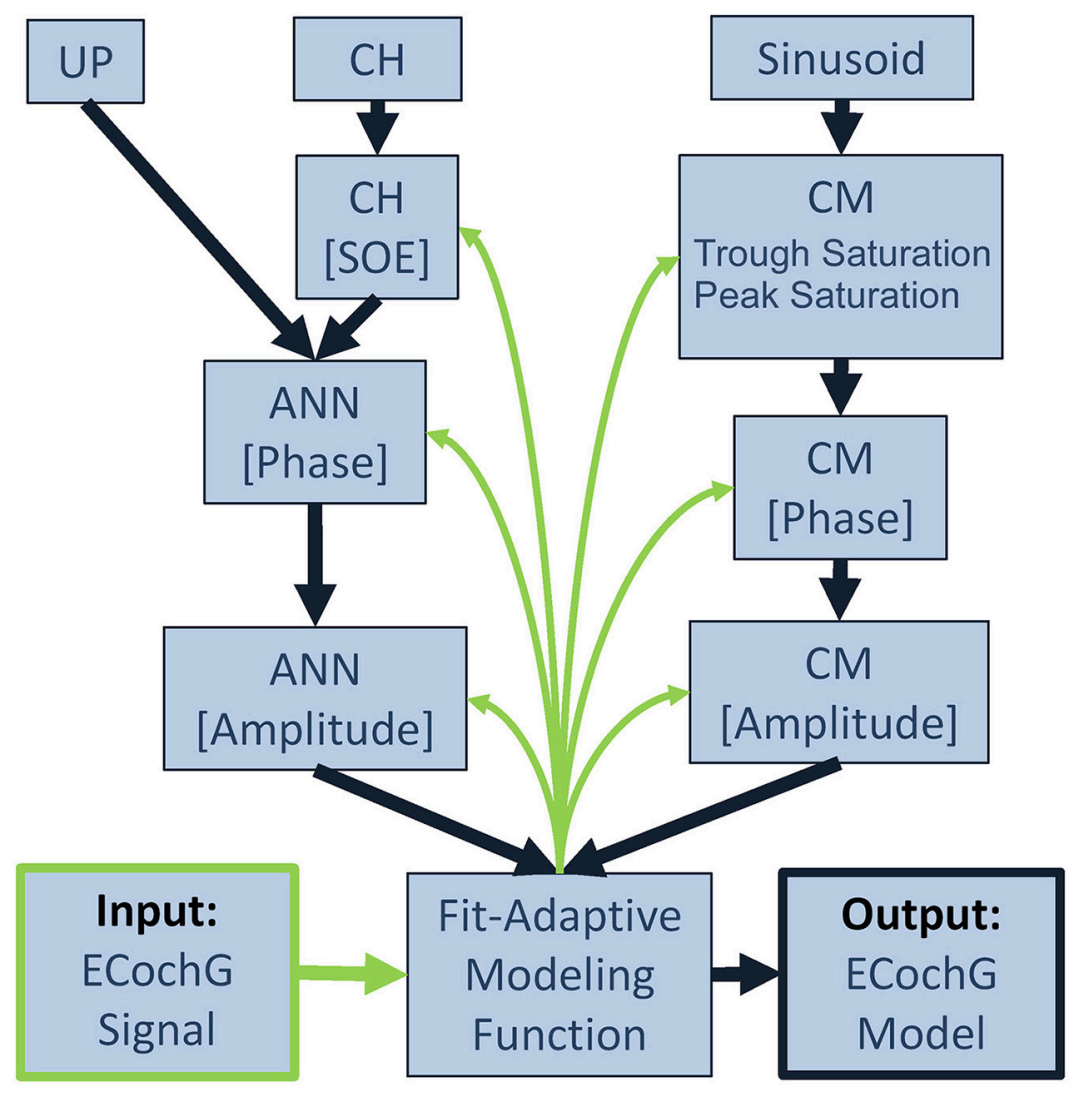
Block diagram for fitting an observed ECochG to model parameters. The ongoing portion of a recorded/input ECochG signal (lower left corner) is the basis for a fit-adaptive modeling function (center, bottom). To estimate the hair cell contribution (right column), the fitting function generates a sinusoidal CM at the stimulus frequency and optimizes the coefficients for amplitude and phase, and saturation of the peaks and troughs of the response. To estimate the neural contribution (left column), a unit potential is convolved with a cycle histogram of variable spread of excitation (SOE) and the resulting ANN amplitude and phase are also optimized. The output of the model is the estimated ongoing ECochG and its associated CM and ANN parameters (lower right corner).

A schematic representation of the analytical process performed by the computational model is shown in Figure 3. To fit an observed ECochG using the model, the averaged ongoing response was evaluated using a nonlinear least squares curve fitting function (MATLAB function “lsqcurvefit”) which calculated optimized values of the CM and ANN parameters (_*A*_*CM*__, _*A*_*ANN*__, φ_*CM*_, _φ_*ANN*__, SOE, peak saturation and trough saturation) based on Equation (4). The specific least-squares algorithm implemented used the “trust-region-reflective” approach because the model was defined with specified equations (Equations 1–4) and the parameters were bounded. Optimized parameters were returned when the output waveform approximated the input signal, using the default optimality tolerance of 1 × 10^−6^.

Goodness of fit was evaluated using regression analysis to calculate the degree of correlation (*r*) and determination coefficient (*r*^2^) between the average cycle of the recorded ECochG and one cycle of the modeled ECochG. Frequency spectra of the modeled ECochG and the individually modeled CM and ANN components were also computed using FFTs.

The model reports the amount of “CM” and “ANN” required to best fit the input waveforms. However, for various reasons described throughout the manuscript these modeled results are not identical to the actual amounts of CM and ANN that produced the waveforms, only an approximation of them. To avoid calling them “mCM” and “mANN” throughout, for example, it should be understood that the reported CM and ANN represent these approximations.

### Generation of simulated signals for model testing

In addition to the human and animal data sets from ECochG, a third data set was a series of simulated signals where the values of each parameter were systematically varied. These simulated signals served to determine the model's ability to detect the changes and observe the effects of the change in each parameter on the others. The simulated signals used the same fitting functions for the CM and ANN as described above.

## Results

### Modeled fits to the average cycles from human CI recipients

**Figure 4:**
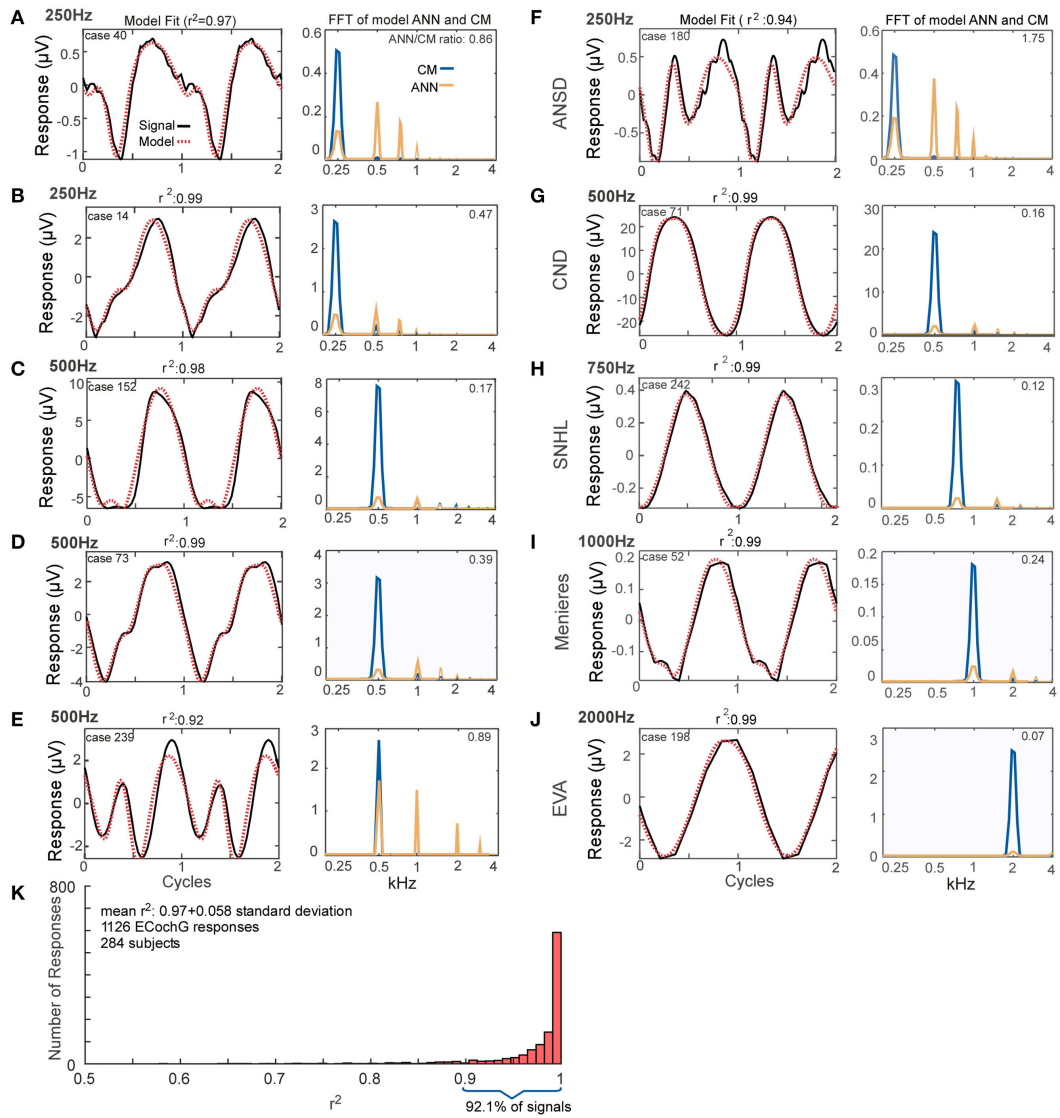
Model fits to ECochG responses in human subjects. **(A–E)** Responses from different subjects to 250 Hz **(A,B)** or 500 Hz **(C–E)** show that the output of the model (left panels, red, dotted line) is able to reproduce the wide variety of waveforms seen in human CI subjects (solid black lines). From the model, the spectra of the CM and ANN used to produce the fit can be produced (right panels). For each case the linear fit between the two curves was described by the *r*^2^ value, and the ANN/CM ratio is given for the spectra. **(F–J)** Similar to the previous examples, except these cases are from subjects with different hearing loss etiologies, to indicate the heterogeneity of causes leading to cochlear implantation (ANSD, auditory nerve spectrum disorder; CND, cochlear nerve deficiency, SNHL, unknown cause of sensineural hearing loss; Meniere's, Meniere's disease; EVA, enlarge vestibular aqueduct). The responses are shown in order of increasing stimulus frequency. The spectrum of the ANN is slightly displaced for clarity. **(K)** Across all recordings (*n* = 1,126) from 284 subjects, the model was able to fit observed ECochG signals with an mean *r*^2^ of 0.97 ± 0.058 (standard deviation).

The fits between recorded waveforms used as inputs and the outputs produced by mixing parameters of the CM and ANN are shown in Figure 4. The examples in Figures 4A–E were chosen to illustrate the variety of waveform morphologies seen to low frequency tones. The waveforms show the inputs and modeled outputs to two concatenated average cycles (left panels), and the spectra show the magnitudes of the individual CM and ANN components (right panels). Some of the responses showed strong distortions compared to the sinusoidal stimuli (e.g., Figures 4A,E), while in others the distortions were smaller (Figures 4B–D). Metrics used to compare the average cycle and model fit were the correlation coefficient (*r*) between the two (from the xcorr function in MATLAB) and the coefficient of determination (*r*^2^). The additional examples in Figures 4F–J show responses and the modeled fits across a wider range of stimulus frequencies (250–2,000 Hz) and in subjects with a variety of hearing loss etiologies. The case shown in Figure 4F, reported as ANSD, showed extreme distortions and a strong ANN to a 250 Hz tone. Another case with a specific type of ANSD, cochlear nerve deficiency (Figure 4G) had very small distortions or ANN, as did a case with an unknown cause of sensorineural hearing loss. Distortions could be present to 1,000 Hz (Figure 4I), while to 2,000 Hz it was absent; in this case there was only saturation (Figure 4J).

Figure 4K demonstrates the distribution of the fits produced by the model based on the analysis of all of the ECochG signals from 284 CI recipients. The mean *r*^2^ produced by the model, based on analysis of 1,241 signals recorded, was 0.97 ± 0.051 (standard deviation).

The data in Figure 4 indicates the model can accurately reproduce the recorded waveforms from CI subjects, and that the ANN/CM ratio reported follows the degree of distortions (other than saturation that can be attributed to the CM) in the waveforms. This data suggests that the model is a plausible means to analyze the responses to assess the underlying sources. We will test this idea with three data sets, first with simulated signal that can be varied parametrically, second with data from gerbils before and after application of neurotoxins to the round window, and finally in the sample population of CI subjects.

### Assessment of the model using simulated signals

**Figure 5:**
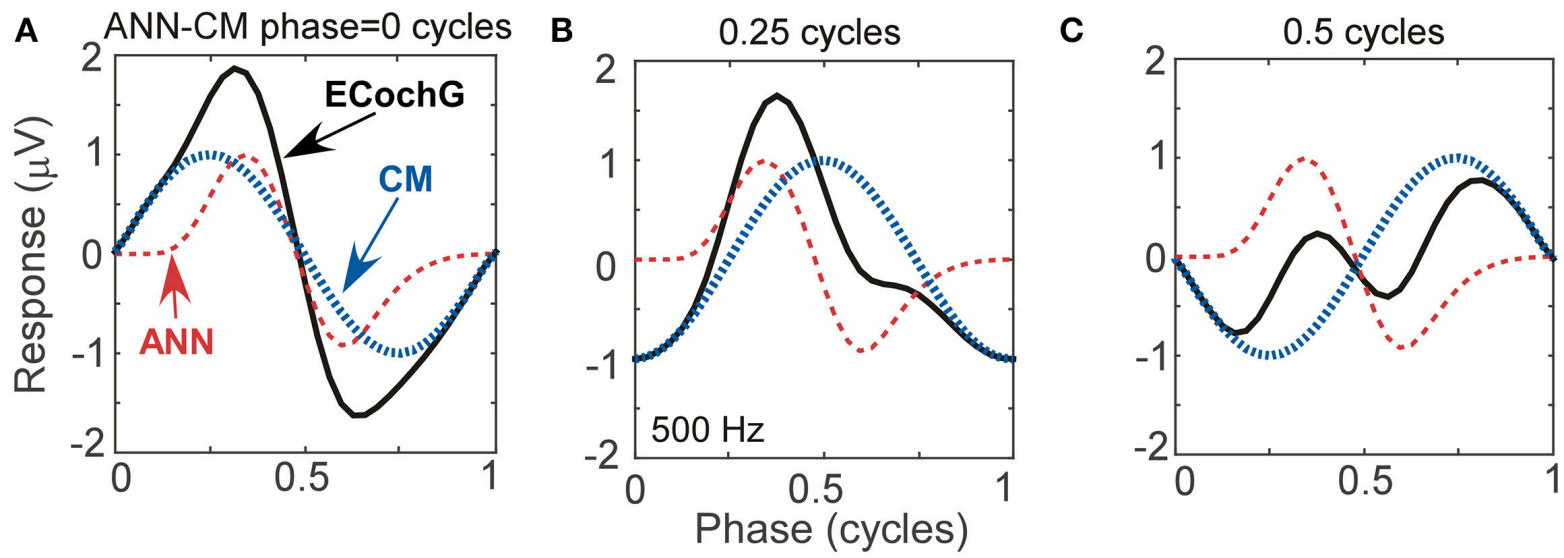
Waveforms generated using simulated signals varied in phase. **(A)** When the CM and ANN are in phase, the waveform is only slightly distorted, and the amplitude is maximal. **(B)** When the CM and ANN are ¼ cycle out of phase, the distortion increases. **(C)** When the CM and ANN are ½ cycle out of phase the distortion is even greater and the overall response magnitude is at a minimum.

To help understand interactions between ANN and CM that help fit particular shapes, and to evaluate possible interactions between parameters returned by the model, we simulated waveforms with parametric variations using the same equations for the CM and ANN that the model used to fit ECochG signals. In Figure 5, we show effects of variation of the phase between the CM and ANN when the amplitudes of each remained the same. This manipulation resulted in waveforms which closely resembled the physiologic signals we have collected from experiments with human CI recipients (see Figures 4E, 4I, and 4E for analogs of Figures 5A, 5B, and 5C, respectively). The phase relationship also changed the overall peak to peak magnitude of the ongoing response, which was at its largest when the two signals were in phase (Figure 5A) and smallest when out of phase (Figure 5C), due to constructive and destructive interference.

**Figure 6:**
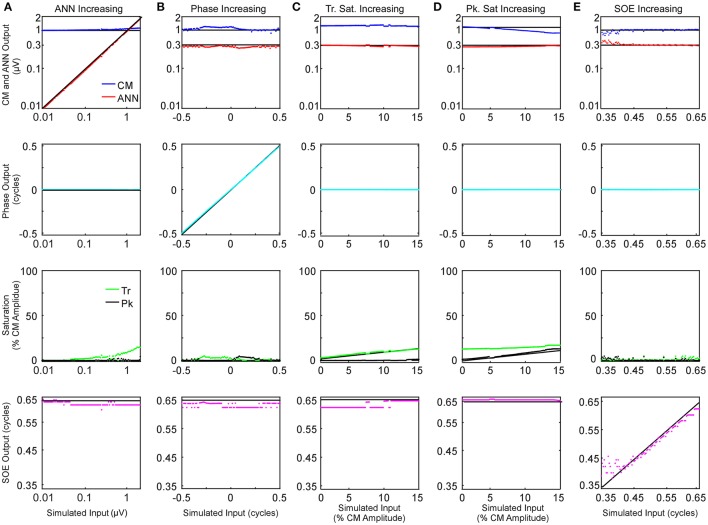
Parametric examination of model outputs to simulated signals. The parameter varied is changed along the columns **(A–E)**, and the responses obtained for each parameter is varied by row. **(A)** The ANN amplitude was gradually increased from 0.01 to 2 μV with CM amplitude of 1 μV, no phase difference between the two signal components or trough or peak saturation, and SOE of 0.65 cycles. **(B)** The phase difference between the two CM and ANN was gradually increased from −0.5 to 0.5 cycle while CM amplitude was 1 μV, no trough or peak saturation, and ANN amplitude was 0.3 μV with SOE of 0.65 cycle. **(C)** The trough saturation of the CM component was varied from 0 to 15% of the CM amplitude with no peak saturation, the ANN amplitude was 0.3 μV in dB and SOE 0.65 cycles while the phase difference between the two signal components was zero. **(D)** The degree of peak saturation of CM was varied from zero to approximately 10% of the CM amplitude of 1 μV while trough saturation was stable at 15% of the CM amplitude; ANN amplitude was 0.43 μV in dB, SOE 0.65 cycles and phase difference between the two components zero. **(E)** The SOE increased from 0.35 to 0.65 cycles while the CM amplitude was 1 μV, ANN amplitude was 0.3 μV and no trough or peak saturation and the phase difference between these two signal components was zero.

The effects of parametric variations of the inputs on the outputs of the model are shown in Figure 6. The parameter that was varied is indicated for each column (Figures 6A–F) and the outputs of the model are shown in the rows. Each panel shows the output to a series of 100 input signals. The input values are indicated by black lines. Only small deviations were seen in the amplitudes of the CM and ANN (**top row**) and the phases between them (**second row**), with the largest deviation occurring to the CM amplitude as symmetric saturation increased (Figure 6D, top row, blue trace). For the trough saturation (third row, green trace) a relatively large deviation occurred as the ANN became large (Figure 6A), but this had only a small effect on the CM amplitude. The peak saturation parameter (third row, black trace) and the SOE, showed small deviations that were associated with minor effects on the CM and ANN amplitudes, and did not affect the phase measurement. These results indicate the model can detect independent parameter changes in the underlying formulae, and that interactions of the parameters do occur, but do not appear to be major.

### Modeled fits of the ECochG signals from gerbils before and after application of neurotoxins

The previous data showed that the model provided good fits to the raw curves and tracks the changes in simulated signals. To further assess how well it could capture the ANN and CM in ECochG responses, experiments using neurotoxins were performed in gerbils. Expected effects of the neurotoxins included (1) a reduced proportion of ANN, (2) little or no effect on the CM, (3) low-pass filtering of the ANN compared to the CM due to the range of phase-locking in auditory nerve fibers, and (4) greater compression of the rate-level function in the ANN compared to the CM; i.e., there should be a greater proportion of ANN to low and moderate intensities than to high intensities in low frequency sounds. These features, if captured by the model, could then be experimentally related to the ANN.

**Figure 7:**
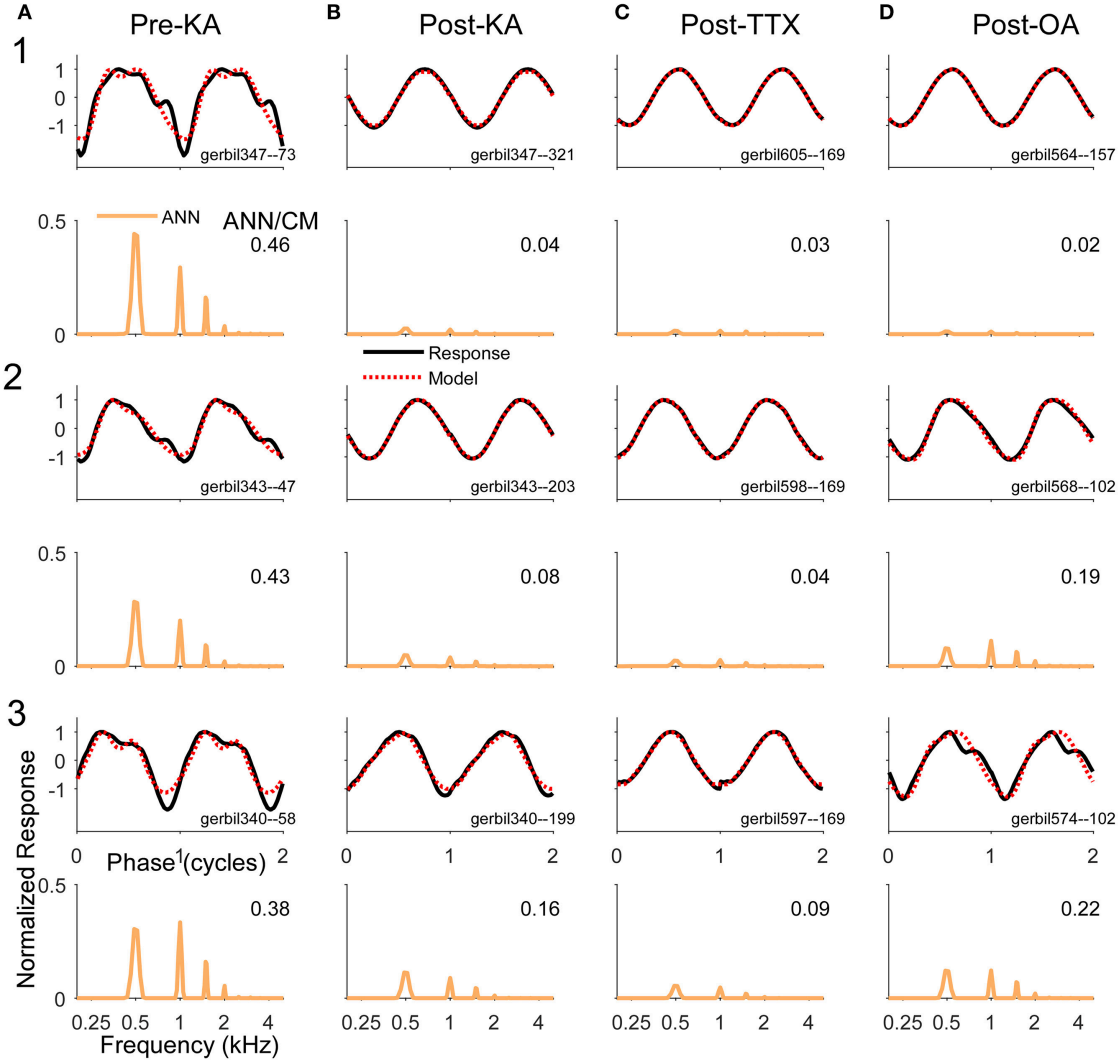
Examples of waveforms and frequency spectra of ECochG signals in response to 500 Hz tone burst at 50 dB SPL **(A)**. Three examples (1–3) recorded prior to KA. The waveforms shown strong distortions and in the ECochG and model waveforms (top panels) and the ANN has multiple harmonics in its spectra (bottom panels). Both sets of data were normalized by the maximum response. The numbers in the spectra represent the ANN/CM ratio. The CM is not shown. **(B–D)** Three examples each (1–3) recorded after KA, TTX, and OA, respectively. The waveforms show less distortion and smaller ANN/CM ratios, although the ANN is not completely removed in most cases. The cases (1–3) are in order of least to most remaining ANN for that drug. The Pre-Drug condition for TTX and OA are not shown, but were similar to that for Pre-KA.

Examples of the effects of the different neurotoxins are shown in Figure 7. The frequency/intensity combination in each response was 500 Hz at 50 dB SPL. This stimulus was chosen for illustration because: (1) the phase-locking is expected to be strong to this low frequency, so a large ANN is expected; (2) the ANN should be proportionally larger compared to the CM than would be the case at higher intensities; and (3) the 500 Hz region is relatively apical in the gerbil cochlea, so it represents a site where the spread of the neurotoxin can be assessed. In addition, 500 Hz is the “sweet-spot” for human CI subjects, where the responses tend to be the largest, so the choice is relevant for our main purpose. The left column shows responses from three gerbils (Figure 7A1–3) prior to any drug application. Each case shows the signal waveform and the model fit (top) and the FFT of the ANN as reported by the model (bottom). Both the waveforms and FFT are normalized by the maximum firing rate. The numbers in the FFTs are the ANN/CM ratio reported by the model. For each neurotoxin (Figures 7B–D), the three examples (Figures 7B–D, 1–3) were chosen to cover the range of distortions remaining; cases in row 1 had the least remaining distortion, those in row 2 an intermediate level, and those in row 3 were at the upper end of distortions seen for that drug. The “Post-KA” responses (Figure 7C) are from the same gerbils as the “Pre-KA” responses (Figure 7A). The main results were that application of the drugs removed most of the distortions compared to the Pre-KA responses, and that the ratio of ANN/CM reported decreased. Application of TTX (Figure 7B) resulted in more complete removal of the distortions and reported reduction in the ANN compared to KA (Figure 7C), or OA (Figure 7D), although with each substance cases with nearly complete reported removal of the ANN occurred (e.g., row 1).

**Figure 8:**
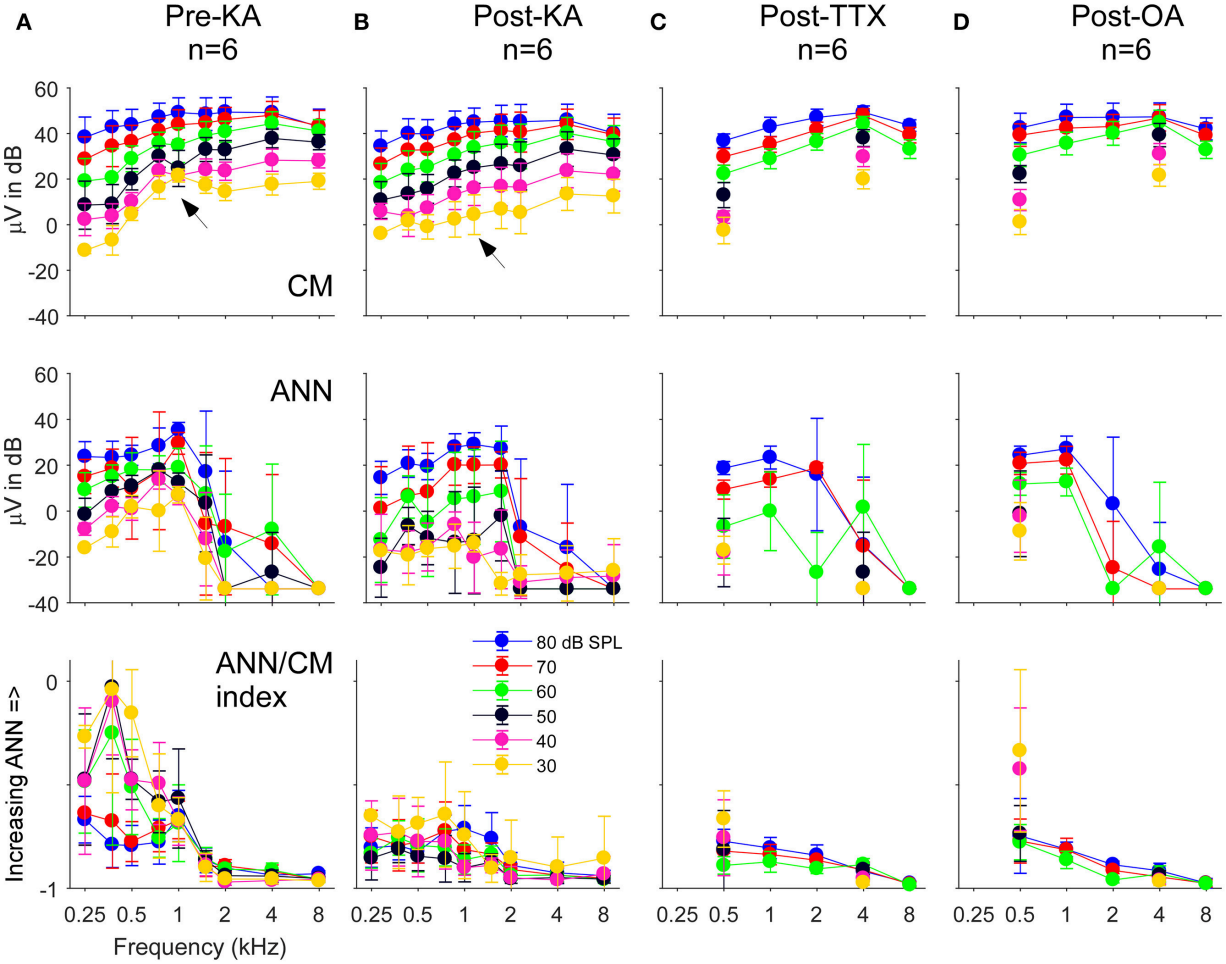
The CM, ANN, and ANN/CM index reported by the model as functions of frequency and intensity. **(A)** The Pre-KA condition. The CM shows an orderly pattern of CM across frequency and intensity, with no cut-off to higher frequencies. The arrow represents a small discontinuity to low frequencies (750 and 1,000 Hz) and intensities (30–50 dB SPL). The ANN shows a low-pass cut-off to frequencies >1,000 Hz. However, a non-linearity was introduced—all responses where the ANN/CM ratio was <5% were considered no response (see Text for further explanation). The ANN/CM index, where no non-linearity was introduced, also showed the low pass cut-off to frequencies >1,000 Hz. **(B–D)** Responses after KA, TTX, and OA, respectively. The Pre-Drug condition for TTX and OA are not shown, but were similar to that for Pre-KA. A smaller range of frequencies and intensities was tested with TTX and OA that with KA. In general, the CM was little affected by the neurotoxin. However, the discontinuity seen in the CM was not present after KA (arrow). The ANN/CM index was also reduced to low intensities, but was already small at high intensities so a change was difficult to detect. The reduction in the ANN and ANN/CM index was greater for KA and TTX than OA. Errors bars are standard deviation.

The population data for the gerbil experiments across frequencies and intensities is shown in Figure 8. The four columns, representing the responses recorded in gerbils before application of any neurotoxin (Figure 8A) and the effects of the drugs (Figures 8B–D) are the same as the previous figure. The rows represent the CM (**top**) and ANN (**middle**) reported by the model which were used to calculate the “ANN/CM index” (**bottom**). The index is an alternate method for reporting the proportion of ANN using the formula (ANN-CM)/(ANN+CM), so that negative values indicate CM larger than ANN (−1 is all CM), 0 indicates equal amounts of CM and ANN, and positive values indicate greater ANN than CM (+1 is all ANN). A larger range of frequencies and intensities was tested in the KA experiments compared to when TTX or OA was used. Across the top row, the use of the neurotoxins had little effect on the CM, although to low intensities in the post KA cases the values reported for 750 and 1,000 Hz were reduced (**arrows**). For the ANN, in the pre-drug condition (Figure 8A) there was a considerable effect of frequency with both the ANN (middle) and the ANN/CM index (**bottom**). This bias of the ANN toward low frequencies is expected from neural phase-locking. However, to achieve this effect in the case of the ANN magnitude the values reported as 5% or less of the total were scored as a zero, because the model rarely produced an ANN much smaller than 5%. Without this cut-off the ANN reported for high frequencies and high intensities was only slightly lower than for low frequencies; i.e., because the responses themselves were so large even a small percentage produced a relatively large ANN. The cut-off did not affect any of the measurements to low frequencies (<= 1,000 Hz) in the pre-drug condition, and the cut-off was not used for the ANN/CM index, so the low pass filtering of the ANN compared to the CM is clear from the model.

In the post-drug conditions (Figures 8B–D), the ANN was reduced compared to the predrug condition, but large values were still reported to high intensities. These large values were probably due to a mixture of two effects. First, the effects of the drug were variable, so some ANN left over after drug application on average is expected. Second, in the post-drug condition the need for the 5% cut-off comes into play for low frequencies as well as high frequencies. The ANN/CM index appeared to capture the effect of the neurotoxins more accurately than the raw numbers. Note that as in the examples presented earlier (Figure 7) the OA had the least effect.

**Figure 9:**
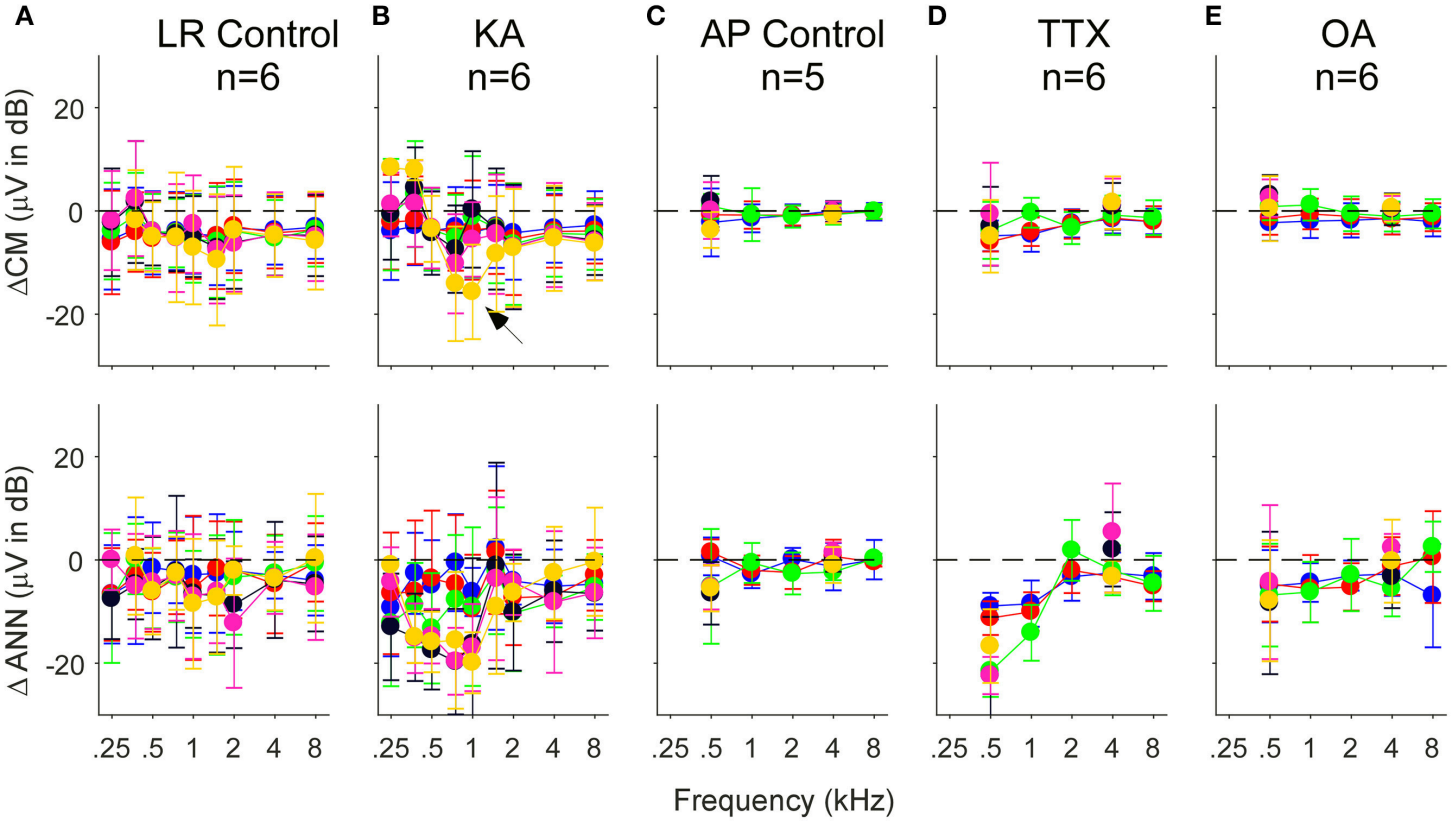
Difference in the CM (top row) and ANN (bottom row) before and after application of vehicle only or vehicle + neurotoxins. Each subtraction is paired between the Pre and Post data for each animal. **(A,C)** Control cases where vehicle only was applied to the round window. For the lactated Ringer's (LR) there was a small reduction in both the CM and ANN that could be related to the passage of time **(A)**. For the artificial perilymph (AP), the smaller frequency, and intensity range decreased the time between recordings, and the reduction in the CM and ANN was smaller **(C)**. **(B,D,E)** Responses after KA, TTX, and OA, respectively. After KA **(B)**, the reduction in the CM to 750 and 1,000 Hz, also shown in the previous figure, was greatest to the lowest intensity (arrow). After TTX **(D)**, the reduction in the ANN was large at 500 and 1,000 Hz, and similar to controls the higher frequencies. After OA **(E)**, the reduction to the lower frequencies was smaller than with KA or TTX. Errors bars are standard deviation.

Another way to assess the effect of the neurotoxin is to compute the difference between the pre and post drug conditions reported by the model. In Figure 9 we show this data for control cases where only vehicle (lactated Ringer's or artificial perilymph) was applied to the round window as well as for when neurotoxins were applied. In the control cases with lactated Ringer's as the vehicle (Figure 9A), a non-specific effect of time is evident by the small decrease in response of the CM and ANN. This is the main reason the frequency and intensity combination were decreased in later experiments. With this smaller stimulus set and change and using artifical perilymph as the vehicle (Figure 9C), the changes in the CM and ANN were much less. After KA (Figure 9B), the subtraction showed the CM to 750 and 1,000 Hz at the lowest intensity (30 dB SPL) to be reduced by a relatively large amount (**arrow**), as shown in the previous figure with the raw data. The CM after KA, TTX, and OA (Figures 9B,D,E) showed no changes in the CM compared to controls. For the KA (Figure 9B) and TTX (Figure 9D), the ANN was reduced to frequencies of 1,000 Hz and below for intensities below 70 dB SPL. To low frequencies at high intensities and for high frequencies the effects of these neurotoxins were small. The ANN showed the greater effect of KA than the CM, with the CM similar to the control. The OA showed the same trends but with smaller effect.

**Figure 10:**
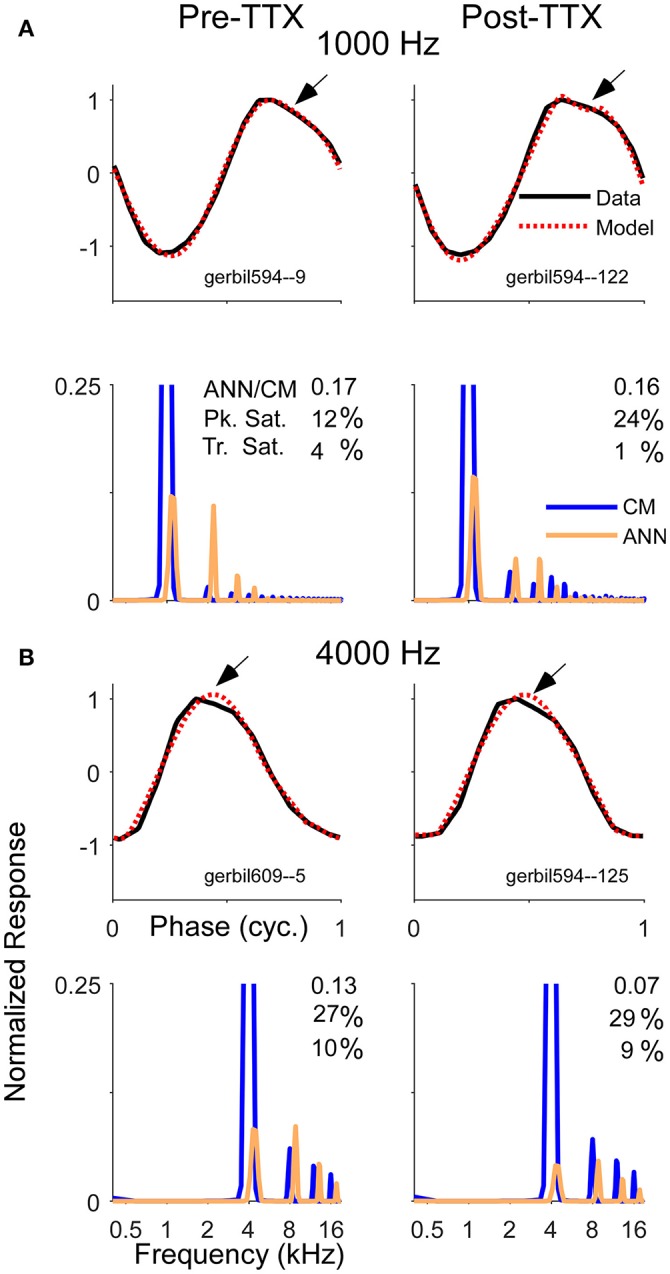
Examples of average cycle waveforms and frequency spectra in response to tone bursts at 80 dB SPL. These examples depict a particular type of ECochG response that does not conform to the shapes expected for CM. To the 1,000 Hz **(A)** and 4,000 Hz stimuli **(B)** there was a sloping response to the clipped peak of the average cycle (arrows). To a 1,000 Hz stimulus at this sound level the ANN should be a relatively small proportion of the response, and smaller still after TTX. For the 4,000 Hz stimulus there should be little or no ANN either before or after TTX. Thus, these waveforms are likely to be nearly-pure CM. The model did capture considerable clipping of the CM, indicated by the large saturation values reported for the peak (Pk. Sat.) and smaller values for the trough (Tr. Sat.). However, the spectrum of each modeled waveform showed considerable ANN even after TTX, suggesting the model interpreted the sloping shape of the CM as ANN. The waveforms and the spectra are normalized to the amplitude of CM contribution measured by the model. The CM of the first harmonic is off-scale to emphasize the higher harmonics, which were present due to the clipping. The spectrum of the ANN is slightly displaced for clarity.

With the KA and the TTX, the reduction of the ANN was less substantial for high than for low intensities, corresponding to the larger remaining ANN to high intensities. However, the expected effect is that the largest reduction in the ANN would be to high intensities, since the neurotoxin would have the greatest effect on the cochlear base, thus blocking spread of excitation. Remaining ANN from the apex would be relatively less affected by the neurotoxin. Thus, less ANN than was actually removed was detected when it is was a small or neligible fraction of the total response at the beginning, and more of the response was estimated to remain than was likely to actually be present. To help understand possible reasons for these results, Figure 10 depicts examples of waveforms and spectra to 1 and 4 kHz before and after the application of TTX, presented at 80 dB SPL. To the 1 kHz tone, some ANN is expected prior to TTX, but at such a high intensity it should be small relative to the CM. After TTX the ANN should be small or negligible. To the 4 kHz tone there should be no ANN either before or after TTX. However, all four of these responses were reported by the model to have considerable ANN—from 7 to 17% of the CM. In addition, all were accompanied by a similar waveform. To be called purely CM, the model expects a sine wave that can be saturated in the peaks and/or troughs. However, responses shown had a declining, rather than purely saturated, response at the peak (arrows). Although many of the pre and post-TTX responses to high frequencies (and post-TTX to low frequencies) had ANN/CM ratios below 0.05, for those that exceeded this cut-off the waveform shape shown here was often encountered.

### The CM and ANN in human CI recipients as determined by the model

**Figure 11:**
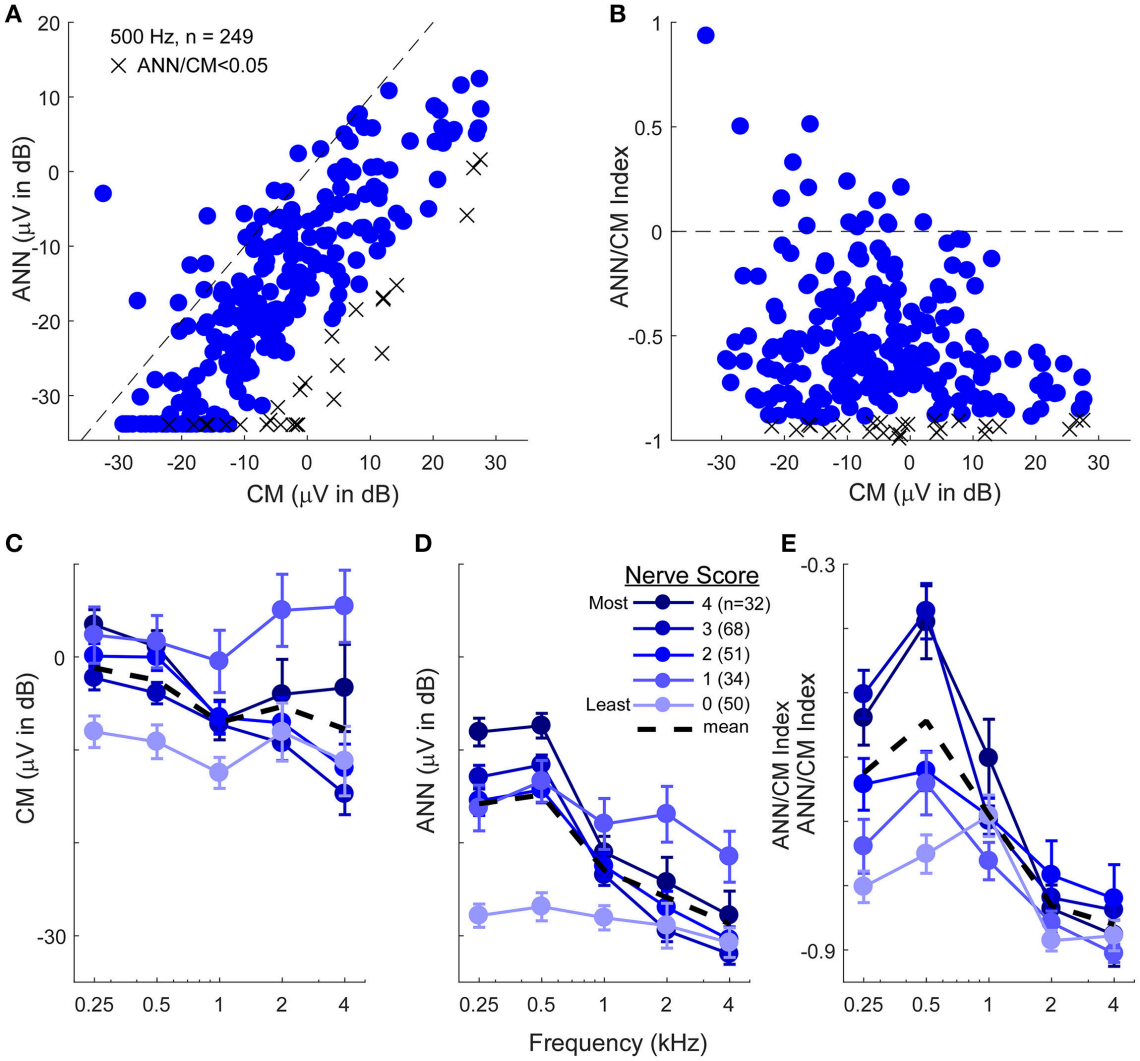
The CM and ANN in human CI subjects. **(A)** In 249 subjects with significant responses (see section Methods) to 500 Hz tone bursts at 90 dB HL the ANN amplitude was generally smaller than the CM (below the line of equality, dashed) but the two were positively correlated (*r* = 0.75, *p* < 0.001). Symbols with an X had an ANN/CM ratio <0.05. **(B)** The ANN/CM index of the same subjects. On this scale an index of−1 is all CM, 0 is equal amount of CM and ANN, and 1 is all ANN. Usually the CM was greater than the ANN, although in a number of cases they were nearly equal, and in a few the ANN was larger than the CM **(C–E)**. The CM **(C)**, ANN **(D)**, and ANN/CM index **(D)** as a function of frequency and with the parameter of “nerve score,” which is a subjective scaling of the neural activity in each cases based on visual observation of the CM and ANN. There was no trend for the subjective nerve activity to reflect the size of the CM, in contrast, the size of the ANN and the ANN/CM index reflected the nerve activity. Both also showed low-pass filtering of similar to that in gerbil. The responses included for each frequency had to be significant (see section Methods) so the numbers of cases differ by a small amount for 250–1,000 Hz (>80% of cases have significant responses to these frequencies) but are fewer to 2 and 4 kHz (43 and 26%, respectively). Errors bars in **(C–E)** are standard error.

The data presented to this point support the ability of the model to reproduce waveform shapes in CI subjects (Figure 4), and the parameters identified provide reasonable estimates of the CM and ANN for most frequency/intensity combinations before and after neurotoxins (Figures 7–10). Here, we apply the model to the population of CI recipients (Figure 11). For 500 Hz stimuli at 90 dB nHL, the magnitude of the reported ANN was typically lower than for the CM. On average, this difference was 14.7 ±13.9 dB (standard deviation). However, there was a general trend for a larger ANN as the CM increased. This trend is expected to the degree that a larger response indicates both larger CM and ANN. However, the data indicated by the “X” symbols are the cases where the ANN/CM ratio was <0.05, and in some of these cases, such as for cochlear nerve deficiency (see Figure 4G), it is highly likely that the ANN would be small or absent. Thus, as with the animal data, the model as currently implemented does not allow for small or absent ANN when the overall response is very large. The average reduction compared to the CM in these cases where the ANN ratio was <0.05 was 26.2 dB, so this appears to be essentially a lower limit for the ANN using the model. Figure 11B shows there was a wide variety in the proportion of the ANN across cases. In the large majority of cases (93%) the ANN/CM index was negative, indicating a predominance of CM over ANN (mean index of −0.56 ±0.31, or an average of about 3.5 time larger CM than ANN). However, a number of cases had an ANN approaching 50% of the CM (index of 0), and in some the ANN contribution was reported as larger than the CM.

To assess the effects of frequency, the ECochG signals belonging to each individual were categorized based on a visual assessment of the neural activity, including evaluation for the presence of a CAP and ANN across the frequency range (see section Methods). The data for the CM was not well-ordered by the amount of neural activity (Figure 11C), and showed only a small frequency effect (these cases show only responses that were significant for each frequency, so the numbers are smaller for 2 and 4 kHz compared to 250–1,000 Hz). In contrast, the reported ANN supported the results of the subjective assessment (Figures 11D,E). As with the gerbil data, a non-linearity at ANN/CM ratio of 0.05 was applied forcing lower ratios to have zero ANN (Figure 11D). The CM/ANN index showed a similar trend as the ANN magnitude without no non-linearity used (Figure 11E). For cases with the highest nerve score the cut-off frequency for the ANN was similar to that seen in the NH gerbils, while the responses in cases with the lowest nerve scores were similar to that seen with gerbils after neurotoxins.

## Discussion

Although, the responses to tones have long been known to contain both CM and ANN, methods to quantitatively separate them have been largely lacking. Here, we created an analytic model of the CM and ANN intended to separate and estimate the magnitudes of these two components of the ongoing response. We used the model to analyze ECochG responses recorded in CI recipients, NH gerbils before and after application of a neurotoxin, and simulated ECochG signals. The model succeeded in capturing the overall shapes of waveforms in CI subjects (Figure 4), was affected in generally predicable ways by parametric manipulation of simulated signals (Figures 5, 6), captured aspects of the responses expected after application of neurotoxins in gerbils (Figures 7–10) and provided estimates of the ANN and CM in human CI subjects that generally matches that of a subjective estimate of neural activity (Figure 11). However, the model also showed limitations, of which the most important was to overestimate the amount of ANN in cases where little or none is expected, such as after neurotoxins or in some CI subjects, and to underestimate the amount of ANN when the CM is extremely large, such as to high intensities in normal hearing animals.

### Need for the model

Masking techniques can reveal the presence of the ANN in many cases, but can quantitatively recover only the amount that is masked, which for suprathreshold stimuli in single unit studies is not the entire neural component (Smith, 1977; Harris and Dallos, 1979). In addition, in CI subjects the stimulus levels are already very high (typically >100 dB peakSPL), so maskers have to be presented at levels that can be prohibitive. In addition, recovery from masking is relatively slow (Snyder and Schreiner, 1985; Verschooten et al., 2015), a major issue with intraoperative techniques. We have tried numerous other methods to quantify the ANN in animals and CI subjects prior to adopting the modeling method used here. As described in Figure 2D, the ANN has inherent asymmetry due to the half-wave rectification of phase-locking in auditory nerve fibers. Thus, the ANN typically contributes a robust 2nd harmonic in the response. This has also been called the “auditory nerve overlapped waveform” (Lichtenhan et al., 2013, 2014). However, the 2nd harmonic is not a quantitative measure of neural contribution because most of the energy of this waveform is periodic at the stimulus frequency, i.e., in the first harmonic, where it is mixed with the CM. The ANN and CM are produced by independent processes that can have different spatial distributions in the cochlea, which results in highly variable phase relationship between the two signals. Therefore, the proportion of ANN present in the first harmonic cannot be predicted by the sizes of the higher harmonics alone. Finally, the second harmonic is not entirely ANN, as high stimulus intensities can cause asymmetric and symmetric saturation of the CM which results in even and odd order harmonics as well (Teich et al., 1989).

In addition to investigating measurements of each harmonic and the total harmonic distortion, we have used cross-correlation and error measures between the average cycle and a sinusoidal representation of the stimulus, as well as shape distortions in the response such as the form factor, crest factor, and skew. The spectral and time-based approaches both identified features indicative of the ANN in many cases, such as the presence of 2nd harmonic, low correlation with a sinusoid, low form factor, high crest factor, or high skew. While these approaches are not quantitative, in most cases their results agreed with our visual assessment of the waveforms. However, with each measure there were clear false positive and false negatives in terms of identifying the degree of ANN, based on visual examination of the average cycle for distortions indicative of neural activity that has been our “gold standard” for identifying the presence of ANN. This visual approach is strongly informed by the animal experiments with neurotoxins, where absence of the ANN was indicated by the loss of the distortions except for saturation that can be attributed to the CM.

It was because of these issues that we considered the approach of using an adaptive model which treats the ECochG waveform as the sum of the discrete CM and ANN signals. This approach depends on accuracy of the equations used to estimate the physiological processes, which we have only partially achieved in this early implementation. Based on our experience up to this point, physiological signals in which the ANN is either very small or exceptionally large relative to the CM are challenging for the model to analyse.

### Basis of the model: the CM

The CM was modeled as a sinusoid with parameters of peak and trough saturation. A benefit of this method is that it requires no a priori knowledge or assumptions about the shape of the function or operating point—the proportion of open channels in hair cell stereocilia in the absence of sound stimulation. In a population response the shape of input/output function will be affected by the spatial extent of responding hair cells which will be stimulated at different effective levels according to their distance from the characteristic frequency locus of the stimulation frequency. In addition, the CM will be a mixture of contributions from outer and inner hair cells, which can have different operating points. By using such a simple and hard-edged description we probably underestimate the complexity of the responses produced by hair cells. In particular, responses in gerbils without ANN, either after neurotoxins or to high frequencies before neurotoxins, show what resemble cycle-by-cycle-adaptation to high intensity sounds (Figure 10). It is not clear what drives this small decline in response during each cycle in some cases. If such adaptation were present in the model it might reduce some of the response interpreted as ANN that is really CM.

### Basis of the model: the ANN

The ANN was modeled as the convolution of the UP and CH, and included a parameter to represent the effect of SOE. This convolution procedure is similar to the convolution of the UP and PST histogram that has been used successfully to model the CAP (Goldstein and Kiang, 1958; Chertoff, 2004) with the cyclic firing to low frequencies in the PST collapsed to produce the CH (Snyder and Schreiner, 1984). After piloting a range of frequencies, the UP was ultimately modeled as a single cycle of an 1,100 Hz sinusoid. The use of a single cycle is similar to the UP determined from experimental data (Versnel et al., 1992b), although we have not yet implemented the exact shape they described. A better approximation of the UP is also an improvement to the model that could be implemented. The shape of the CH was modeled as a stretched lognormal probability density equation, with the variable width of the curve (σ) representing the SOE. These equations represent a version of the underlying processes, and a more accurate description of the actual physiology is likely to be achieved if a biophysically-based model were used (Carney and Yin, 1988; Meddis, 1988; Meddis et al., 2013; Zilany et al., 2014).

### Results with the model: simulated signals

With simulated waveforms as inputs the model was able to reproduce the values of the parameters across the range encountered physiologically. This simulation was presented in detail to 500 Hz, since that is a frequency where both the CM and ANN can have a wide range of relative values. The features reproduced with the most accuracy were CM amplitude, ANN amplitude, and the phase difference between them. The model reported a small degree of primarily saturation, primarily in the trough, when the ANN amplitude exceeded the CM amplitude. This deviation was accompanied by small deviations in the reported CM and ANN amplitudes. The model was less precise with its estimation of SOE, however, inaccuracies in that parameter did not seem to affect other parameters of the ANN component.

One purpose in using the simulated signals was to assess the effects of phase differences between the ANN and CM on the ECochG waveforms and compare them to the distortions commonly seen in the human and gerbil data. We found that manipulating the phase resulted in a variety of waveforms which closely resembled the physiologic signals we have collected from experiments with the animal model and human CI recipients. The phase relationship also changed the magnitude of the ongoing response, which was at its largest when the two signals were in phase and smallest when out of phase; i.e., there was constructive and destructive interference. This effect has implications for studies of ECochG as a monitoring tool for cochlear trauma during CI surgery. Many of these studies use 500 Hz tones as a stimulus, and some monitor the magnitude of the response, either as an RMS signal (Campbell et al., 2015, 2016) or as the peak of the spectrum at the stimulus frequency (Koka et al., 2016). Because of the expected effect of phase interactions, which was demonstrated here in the model, in the past we (Fitzpatrick et al., 2014; McClellan et al., 2014; Formeister et al., 2015) and others (Dalbert et al., 2016) have summed the peaks of the spectrum of the response to each stimulus frequency as the measure of response magnitude. By summing the spectral peaks, rather than calculating their RMS value as would be done to reproduce the time waveform, the contributions of the distortions to the overall signal are given more weight. While summing rather than squaring the response peaks partially mitigates the effect of phase when assessing the magnitude of the ECochG response, the model offers the possibility of measuring the potentials separately and thus accurately measuring the overall response independent of phase effects.

### Results with the model: studies using gerbils

The results from the gerbil indicate that the model captures some important features of phase-locking in the auditory nerve across frequency and intensity. It reports a larger CM than ANN, with the major effects of neurotoxins limited to the ANN. In the case of KA we did see some effect of KA on the CM at a few frequency/intensity combinations, but this was not seen with the other neurotoxins. However, the vehicle was also different between the experiments (lactated Ringer's for KA and artificial perilymph for the others) so it hard to know what to attribute this difference to. The proportion of the ANN relative to CM is strongly reduced to high frequencies compared to low, with the cut-off between 1,000 and 2,000 Hz, consistent with the range where phase-locking in gerbil auditory nerve fibers has the greatest synchrony (Ohlemiller and Siegel, 1998; Versteegh et al., 2011). The relationship with intensity is similar to that expected from compression of the ANN relative to the CM, which is that the proportion of ANN is much greater to low intensities compared to high. Thus, the model does identify the major features of phase-locking expected from single unit studies and extrapolated to a population response.

The major limitation in the model was the report of substantial ANN in cases where little or no neural responses were expected (e.g., high frequency stimulus, or after treatment with a neurotoxin). Large values of ANN were reported when the CM was large, even if the overall percentage reported was relatively low. To help mitigate this error, we set values of ANN to be zero when the ANN/CM ratio was <0.05. There is evidence (Figure 10) that the flaw lies in an incomplete modeling of processes which can affect the CM waveform morphology. A promising direction is to allow some adaptation in the response on a cycle-by-cycle basis. The model also struggled with some responses to low frequencies presented at low to moderate intensities—these signals tended to have the largest ANN and produce highly complex waveforms. While the model accurately identified large ANN amplitude in these cases, the correlations between the input and the model signals tended to be lower than the average, suggesting possible areas of improvement in the implementation of UP, CH, and SOE.

Application of KA also resulted in a small decline of the CM signal magnitude to low frequencies (750 and 1,000 Hz) and intensities (30 dB SPL), suggesting the neurotoxin affected hair cells, or that the model was incorrectly assigning some of the ANN to the CM prior to KA application. A similar change in the CM did not happen with either TTX or OA. A small effect of KA on the CM has previously been reported in other animal models (Zheng et al., 1996; Sun et al., 2001). In addition, although we have not examined the question in detail, some effect on the CM, either an increase or decrease, can be expected in individual cases due to changes in the efferent system that can affect the operating point of outer hair cells. Such changes are expected once the afferent input is removed, but the direction may vary across cases.

The frequency range of ANN reported by the model is a close match to the range where the ANN was detected in a spectral analysis using some of the same KA data (Forgues et al., 2014). It is also similar to the range of the “auditory nerve overlapped potential,” reported in similar experiments in other species (Lichtenhan et al., 2013, 2014). In contrast to the evoked potential results, single units in gerbils can show phase-locking to frequencies up to 3–4 kHz (Versteegh et al., 2011), as is also reported in other species (Johnson, 1980; Weiss and Rose, 1988). There are at least two reasons why the ANN in ECochG recordings may have a more limited phase-locking range than the single units. The first is that the ANN may only be detectable over the range of phase-locking where the synchrony is the highest. In gerbils and most species there is a steep decline in the vector strengths of single units beyond about 1,000 Hz. The second is that there will also be low-pass filtering of the ANN due to the overall UP duration of ~1 ms (~period of 1,000 Hz sinusoid), as previously suggested by Lichtenhan et al. (2013). Due to the UP's relatively long duration, overlapping responses to higher frequency stimuli may reduce the cyclic component in the evoked response.

A main assumption of the model is that the ongoing response consists of only the ANN and CM. This misses at least one known source of cochlear electrical responses—the dendritic current that is produced from the sum of synaptic currents in auditory nerve fiber terminals (Dolan et al., 1989). Since the dendritic potential is not based on spikes, the correlate of the UP would be the synaptic EPSP from transmitter-gated channels. TTX blocks only the action potentials and should not affect these EPSPs, unlike KA which removes the nerve terminal, and OA which prevents further depolarization. This dendritic current is not currently considered in the model. By initial application of TTX followed by KA, the dendritic contribution can be isolated as the difference of the response seen after each compound. Preliminary results from this experiment show the dendritic response to be present but smaller than the spiking component. Future iterations of the model will need to consider both sources of neural contributions to the ongoing response to better account for recorded waveform shapes.

Finally, the model does not include separate functions for inner and outer hair cells. This is reasonable given that the recordings from the round window are the sum of all contributions to the CM, which include both types of hair cells. However, it would be important to know whether the asymmetries are different in the two cell types, which could also be approached pharmacologically in gerbils, as it has in guinea pigs (van Emst et al., 1995, 1996).

### Results with the model: human CI subjects

The results of model analysis of the signals recorded in human CI subjects are encouraging, however, issues similar to those in the animal experiments were present. The reported CM was on average larger than the ANN, by 26 dB on average. This corresponds with our expectation that the ECochG responses in CI subjects are dominated by the CM, which is the reason why the measure of “total response” (sum of all significant responses to harmonics 1–3 across a range of tone burst frequencies) account for more of the variance in outcomes in adults (>40%, Fitzpatrick et al., 2014; McClellan et al., 2014) and in older children (>30%, Formeister et al., 2015) than does audiometric or biographic data (Lazard et al., 2012). That is, the proposed explanation for correlation of outcomes with a signal dominated by the CM in these studies is that the degree of hair cell survival is a better correlate to “cochlear health” than is the degree of intact connections with nerve fibers. Here, the CM did not show a low-pass cut-off frequency, consistent with the animal data and basilar membrane movement. Furthermore, it was not correlated with the degree of neural activity determined subjectively, and which was a good fit with the results for ANN, further supporting the view that the CM and ANN in CI subjects do not provide identical information regarding outcomes.

In the population-wide results, as in the gerbil data, the model did not always report a small ANN for cases where the CM/ANN ratio was small; instead, enough ANN was reported for it to scale with the size of the CM. As was discussed with the gerbil results, it may be that the shape of the CM is more complex than a sinusoid with parameters of asymmetric and symmetric saturation, such that any waveform abnormalities beyond those would likely be attributed to the ANN. The importance of this issue is that to the degree the reported ANN is covariant with the CM rather than independent, its value as a independent predictive measure for speech perception outcomes with the CI recipients is limited.

Unlike gerbils, the phase-locking range in the human auditory nerve is unknown. There are some indications that human phase-locking could go to higher frequencies than found in animal single unit studies (Moore et al., 2006), but the more general view is that the weight of evidence supports a range of up to about 1.5 kHz for strong phase-locking, i.e., similar to other species (Joris and Verschooten, 2013). Here we are able to report that the frequency range of the ANN estimated by the model (and seen visually in the average cycle) is similar to that in the gerbil.

## Conclusion

A model based on an analytic description of hair cell and neural contributions to the ongoing responses to low frequency tones was used to separate the ECochG signals into their individual components. This analytical tool can help characterize the residual physiology CI recipients, and can be useful in other clinical settings where a description of the cochlear physiology is desirable.

## Author contributions

TF led the conception of the work under the guidance of DF. TF lead the development of the computational model with guidance of CG and DF. TF and DF jointly designed the experiments performed including simulated signals, signals recorded in animal model and signals from human subjects. TF developed the program which created the simulated signals and personally created each series of signals that were analyzed. TF, CG, and DF each participated in the collection of the animal and human data. TF and DF both worked extensively on analysis and interpretation of the data. TF and DF jointly led the formulation of the initial draft of the manuscript. TF, CG, and DF all worked to continuously develop and revise all parts of the critically important content to produce the final version for submission. TF, CG, and DF give their full permission for publication of the submitted work. TF, CG, and DF all agree to be accountable for all aspects of the submitted work and stand behind its integrity. Should any questions or issues arise, the authors will work proactively to ensure their appropriate investigation and resolution.

### Conflict of interest statement

DF has consulting arrangements and research projects with MED-EL, Cochlear Corp, and Advanced Bionics. The other authors declare that the research was conducted in the absence of any commercial or financial relationships that could be construed as a potential conflict of interest.
